# Incidental Finding of a Low-Grade Appendiceal Mucinous Neoplasm: A Case Study

**DOI:** 10.7759/cureus.77427

**Published:** 2025-01-14

**Authors:** William Olive, Haleigh Isanhart, Joseph Thurman

**Affiliations:** 1 Surgery, Lincoln Memorial University-DeBusk College of Osteopathic Medicine, Knoxville, USA; 2 Surgery, The University of Tennessee Health Science Center, Memphis, USA; 3 Surgery, Fort Sanders Regional Medical Center, Knoxville, USA

**Keywords:** appendiceal, appendix, lamn, mucinous, neoplasm

## Abstract

Low-grade appendiceal mucinous neoplasms (LAMNs) are rare benign tumors of the appendix with possible life-threatening complications. A 25-year-old female patient presented to the emergency room with abdominal pain with symptoms concerning for acute appendicitis. After imaging, it was decided to proceed with an appendectomy. Final pathology showed low-grade appendiceal mucinous neoplasm. Due to their ability to mimic appendicitis, it is important to include LAMNs in the differential diagnosis for patients of all ages, as they can cause life-threatening pseudomyxoma peritonei (PMP). Although this case report highlights a rare condition, it should be on a general surgeon's differential to avoid potential long-term malignancies.

## Introduction

Rokitansky first described an appendiceal mucocele in 1842 [1]. Low-grade appendiceal mucinous neoplasms (LAMNs), a subtype of appendiceal mucinous neoplasms, are rare and, in most cases, incidentally discovered appendiceal tumors that can lead to dire complications if left untreated [2].

Since many cases of LAMNs are asymptomatic, underreporting of incidence is likely. Based on current research, LAMNs are four times more likely to occur in women than in men. They are most commonly discovered in individuals over the age of 50, typically in the sixth decade of life [3]. LAMNs are often asymptomatic, and since they are slow-growing tumors, diagnosis is most likely delayed.

LAMNs, like other gastrointestinal tumors, are positive for the tumor markers CEA and CA19-9, with higher plasma levels being associated with higher levels of dysplasia, which can be used to monitor for recurrence [4]. On histology, LAMNs are seen as mucinous epithelium with low-grade dysplasia. Additionally, LAMNs are confined to the muscularis propria and efface the lamina propria. A key histological finding of LAMNs is that they do not infiltrate surrounding structures. The confined "pushing" pattern can mimic the gross findings of the diverticulum. However, their architecture can appear as villiform, undulating, or flat. LAMNs are classified and staged based on TNM criteria [5].

The clinical presentation for LAMN greatly varies and can even be asymptomatic only to have been found incidentally on imaging for an unrelated concern. When symptoms do present, they often mimic appendicitis; however, other non-specific symptoms have been reported, such as abdominal pain, palpable abdominal mass, changes in bowel habits, obstruction, gastrointestinal bleeding, weight loss, hematuria, and hydronephrosis [6].

Incidental findings of LAMNs can be discovered by endoscopy, radiography, ultrasound, CT, or MRI. On endoscopy, LAMNs can be seen as an erythematous soft cecal mass with a central crater that produces mucin. Plain radiographs can show curvilinear calcifications of the right iliac fossa as well as a mass effect on the bowel, whereas a barium enema will show non-filling of the appendix. LAMNs appear on ultrasound as an encapsulated cystic lesion with an internal onion-skin appearance. CT of a LAMN will show an encapsulated smooth-walled enhancing lesion. Lastly, on MRI, LAMNs, as well as other mucinous neoplasms, appear as hyperintense tubular areas of distention of the appendix on T2-weighted imaging. While these scans can uncover a LAMN, a diagnosis of LAMN can only be determined by histological examination [3].

Treatment of LAMNs is controversial and currently based on their TNM staging. Treatment options include appendectomy for tumors graded a T3 with no other risk factors and a hemicolectomy for tumors graded a T4a with no other risk factors. Risk factors included T stage, appendix perforation, presence of acellular mucin on the serosa, positive surgical margins, and increased risk for recurrence and pseudomyxoma peritonei (PMP). In T4b disease states or in patients with risk factors, cytoreductive surgery and hyperthermic intraperitoneal chemotherapy are indicated [2].

Despite the rarity of LAMN accounting for less than 1% of appendectomy specimens, the complications that can arise from being untreated can lead to disastrous effects for the patients [7]. One of the more problematic complications is pseudomyxoma peritonei. If a LAMN ruptures and spreads to the peritoneum, the mortality rate increases substantially, with a five-year survival rate of 25% [2]. It is important that surgeons strive for negative margins when resecting LAMN; otherwise, a right hemicolectomy might be needed to improve long-term outcomes [2]. Due to the severe complications that can arise from this neoplasm, it is imperative that physicians are aware of its existence and keep this diagnosis in mind when creating a differential for patients. Herein, we present a unique case of a 25-year-old female patient who presented with symptoms of appendicitis to the emergency department only to uncover the source of her symptoms was due to a LAMN.

## Case presentation

A 25-year-old female patient presented to the emergency department complaining of abdominal pain with diarrhea, nausea, vomiting, chills, and dizziness that began that morning. She additionally endorsed pain with urination. She denied any recent history of trauma, chronic illness, or changes in bowel function. The patient denied having any history of abdominal surgeries. Physical examination revealed diffuse abdominal tenderness. The differential diagnoses considered included but were not limited to appendicitis, small bowel obstruction, mesenteric ischemia, infected ureteral stone, and intra-abdominal abscess.

**Figure 1:**
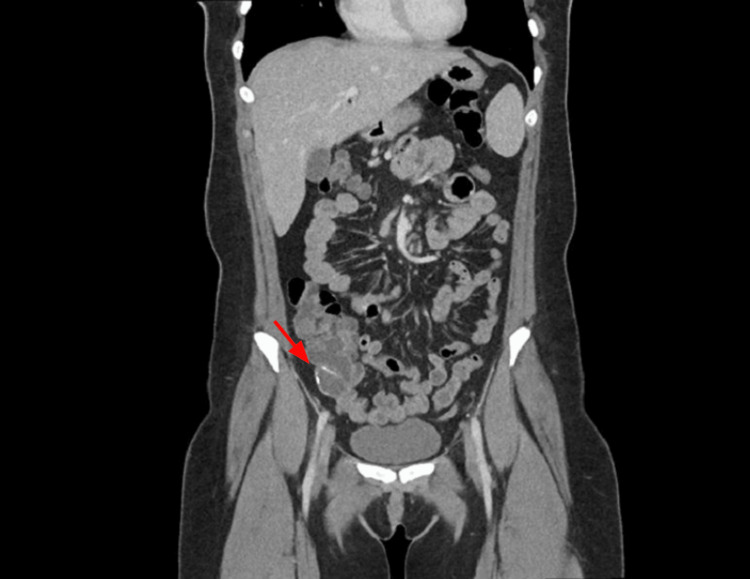
Coronal slice showing the appendiceal calcifications (red arrow)

Laboratory values of the complete hemogram were within normal limits. An abdominal CT revealed an appendix dilated to 2.4 cm near its base, containing curvilinear calcifications in the wall, and there was still communication with the distal appendix (Figure 1). We proceeded with a laparoscopic appendectomy with three ports. Under laparoscopic visualization, the distal third of the appendix was substantially dilated and somewhat firm, but with no obvious serosal abnormality. A stapler was used to transect the base of the appendix and the mesoappendix The appendix was then placed in an endo-catch bag and removed through the umbilical incision.

The appendix was sent for histopathology examination. Pathology slides confirmed a tumor in the distal half of the appendix. It was determined to be a low-grade appendiceal mucinous neoplasm with a grade of G1, well differentiated. The surgical margins were negative, and there was no evidence of perforation or acute appendicitis. The patient followed up with the surgeon in the clinic two weeks later with a standard post-operative visit.

## Discussion

This case report details a rare presentation of low-grade appendiceal mucinous neoplasm (LAMN) in a 25-year-old female patient, an unusual finding given that LAMN typically presents in older adults, often in their sixth decade of life [3]. The patient's presentation with abdominal pain, diarrhea, nausea, vomiting, chills, and dizziness was initially suggestive of acute appendicitis, which led to the decision to perform an appendectomy. Other possible symptoms include a palpable abdominal mass, changes in bowel habits, obstruction, gastrointestinal bleeding, weight loss, hematuria, and hydronephrosis.

Appendiceal tumors are rare, accounting for only 0.5% of gastrointestinal neoplasms; however, 50% of these tumors are identified as LAMNs, which is a cause for concern due to it being a precursor for pseudomyxoma peritonei, an intraperitoneal accumulation of mucus, which can be fatal [8]. LAMNs have a high risk of recurrence, increasing the risk of developing PMP in the future, making proper surgical techniques crucial. LAMNs can be monitored for recurrence using CEA and CA19-9 plasma levels. According to recent literature, less than 1% of appendiceal specimens are diagnosed as LAMN, highlighting the rarity of this condition.

Appendiceal tumors are separated into neuroendocrine (producing serotonin) and epithelial. Morphologically, LAMNs are mucinous neoplasms of the epithelium with low-grade cytologic atypia with effacement of the lamina propria and confined by the muscularis propria but without invasive destruction of surrounding structures [5]. High-grade appendiceal mucinous neoplasms (HAMNs) are also non-invasive but have more dysplastic cells in the mucin compared to LAMNs [9]. Other epithelial tumors of the appendix include adenomas and adenocarcinomas, which, while they can be mucinous, are differentiated by invasion past the epithelial layer [10]. In our case, histopathology revealed a well-differentiated G1 neoplasm confined to the distal appendix with negative surgical margins. These findings are consistent with the typical histological characteristics of LAMN.

## Conclusions

In summary, this case of LAMN in a 25-year-old female patient highlights the importance of considering appendiceal neoplasms on differential diagnoses, even if typical appendicitis symptoms are present.

The majority of LAMN cases present incidentally or with symptoms mimicking acute appendicitis, as seen in our patient. This case of a 25-year-old was particularly notable. Given her young age and the well-differentiated, low-grade nature (G1) of the tumor, appendectomy was an appropriate treatment compared to hemicolectomy or other surgical options, with a low likelihood of recurrence.
